# Systems biology driving drug development: from design to the clinical testing of the anti-ErbB3 antibody seribantumab (MM-121)

**DOI:** 10.1038/npjsba.2016.34

**Published:** 2017-01-05

**Authors:** Birgit Schoeberl, Art Kudla, Kristina Masson, Ashish Kalra, Michael Curley, Gregory Finn, Emily Pace, Brian Harms, Jaeyeon Kim, Jeff Kearns, Aaron Fulgham, Olga Burenkova, Viara Grantcharova, Defne Yarar, Violette Paragas, Jonathan Fitzgerald, Marisa Wainszelbaum, Kip West, Sara Mathews, Rachel Nering, Bambang Adiwijaya, Gabriela Garcia, Bill Kubasek, Victor Moyo, Akos Czibere, Ulrik B Nielsen, Gavin MacBeath

**Affiliations:** 1 Merrimack Pharmaceuticals, Cambridge, MA, USA; 2 Celgene, San Francisco, CA, USA; 3 PatientsLikeMe, Cambridge, MA, USA; 4 Synlogic, Cambridge, MA, USA; 5 L.E.A.F. Pharmaceuticals, Radnor, PA, USA; 6 Torque, Cambridge, MA, USA

## Abstract

The ErbB family of receptor tyrosine kinases comprises four members: epidermal growth factor receptor (EGFR/ErbB1), human EGFR 2 (HER2/ErbB2), ErbB3/HER3, and ErbB4/HER4. The first two members of this family, EGFR and HER2, have been implicated in tumorigenesis and cancer progression for several decades, and numerous drugs have now been approved that target these two proteins. Less attention, however, has been paid to the role of this family in mediating cancer cell survival and drug tolerance. To better understand the complex signal transduction network triggered by the ErbB receptor family, we built a computational model that quantitatively captures the dynamics of ErbB signaling. Sensitivity analysis identified ErbB3 as the most critical activator of phosphoinositide 3-kinase (PI3K) and Akt signaling, a key pro-survival pathway in cancer cells. Based on this insight, we designed a fully human monoclonal antibody, seribantumab (MM-121), that binds to ErbB3 and blocks signaling induced by the extracellular growth factors heregulin (HRG) and betacellulin (BTC). In this article, we present some of the key preclinical simulations and experimental data that formed the scientific foundation for three Phase 2 clinical trials in metastatic cancer. These trials were designed to determine if patients with advanced malignancies would derive benefit from the addition of seribantumab to standard-of-care drugs in platinum-resistant/refractory ovarian cancer, hormone receptor-positive HER2-negative breast cancer, and EGFR wild-type non-small cell lung cancer (NSCLC). From preclinical studies we learned that basal levels of ErbB3 phosphorylation correlate with response to seribantumab monotherapy in mouse xenograft models. As ErbB3 is rapidly dephosphorylated and hence difficult to measure clinically, we used the computational model to identify a set of five surrogate biomarkers that most directly affect the levels of p-ErbB3: HRG, BTC, EGFR, HER2, and ErbB3. Preclinically, the combined information from these five markers was sufficient to accurately predict which xenograft models would respond to seribantumab, and the single-most accurate predictor was HRG. When tested clinically in ovarian, breast and lung cancer, HRG mRNA expression was found to be both potentially prognostic of insensitivity to standard therapy and potentially predictive of benefit from the addition of seribantumab to standard of care therapy in all three indications. In addition, it was found that seribantumab was most active in cancers with low levels of HER2, consistent with preclinical predictions. Overall, our clinical studies and studies of others suggest that HRG expression defines a drug-tolerant cancer cell phenotype that persists in most solid tumor indications and may contribute to rapid clinical progression. To our knowledge, this is the first example of a drug designed and clinically tested using the principles of Systems Biology.

## Introduction

The development of a new drug from original idea to commercial^1,2^ product is a complex process that can take up to 15 years and cost in excess of $1 billion.^3^ Traditionally, drug discovery has been a linear endeavor, progressing from target identification and validation to therapeutic lead identification, often driven by high-throughput screening. The final clinical lead molecule usually emerges after many rounds of trial and error and after numerous elimination steps, rather than as the result of specific design criteria and the subsequent engineering of a therapeutic agent that meets these pre-set specifications.

Initially, intentional drug discovery in cancer was focused almost exclusively on targeting DNA synthesis and cell division, resulting in antimetabolites (e.g., 5-fluorouracil), DNA alkylating agents (e.g., cyclophosphamide), and microtubule stabilizers (e.g., taxanes). These drugs showed efficacy, but at the price of high toxicity due to lack of selectivity.^4^ The identification of cancer-causing genes in the early 1980s started a new era of cancer drug discovery: the development of targeted therapies in the form of monoclonal antibodies and small molecule inhibitors. Two prominent and much-cited examples are trastuzumab (Herceptin) and imatinib (Gleevec). Trastuzumab was the first targeted therapy for use in women with metastatic breast cancer who have tumors that overexpress HER2,^5^ whereas imatinib was approved for the treatment of chronic myelogenous leukemia and is the first selective inhibitor of the Abl kinase.^6^ These notable successes validated the use of a tumor’s genetic makeup to guide the development and clinical use of targeted therapies and provided impetus for the cancer genomics revolution. Large-scale sequencing efforts enabled the identification of numerous genomic alterations and highlighted many new potential targets for cancer therapy and their associated predictive biomarkers.^7^ Unfortunately, not all new discoveries were met with the same success. We soon learned that there is not always a clear one-to-one association between genetic alterations and effective new cancer treatments. Researchers often struggle to understand the functional importance of mutations, a challenge that is further compounded by their heterogeneous distribution in tumors. Consequently, the number of patients with tumors that are dependent on a single oncogenic driver is low. In fact, driver mutations often do not translate across different indications. For example, melanomas with *BRAF* mutations respond well to B-Raf inhibitors, but the same is not true in colorectal cancer.^8,9^

Perhaps the greatest challenge underlying the effective treatment of cancer is that tumors continuously adapt to their environment, evolving ways to circumvent the agents intended to kill them. In order to treat and eventually cure cancer, we need to gain a systems-level understanding of tumors, including how they interact with their microenvironment, how they respond to the immune system, how they evade therapeutic intervention, and how they evolve over time. Only computational methods will allow us to master this complexity and design appropriate strategies to defeat the ‘Emperor of all Maladies’.^10^ To date, Systems Biology has focused mainly on understanding how tumor cells process external signals, with the goals of understanding key pathways that are frequently dysregulated in cancer^11,12^ and of designing novel therapies to block these pathways.^13–15^ Only recently has Systems Biology been used to understand better the spatial and temporal heterogeneity in patient tumors in order to rationally design combination therapies.^16,17^ Clearly, we have a long way to go, but even now we can start to apply these methods to the discovery and development of novel therapeutics.

**Figure 1:**
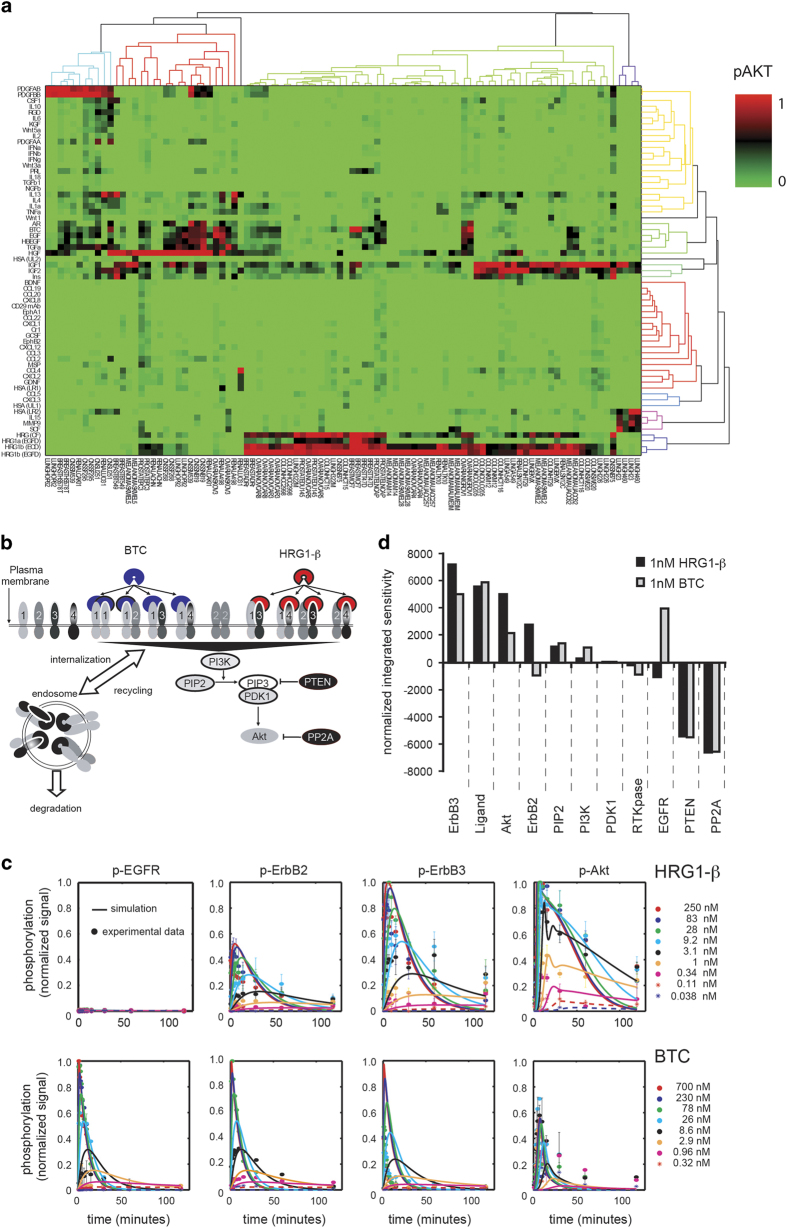
Computational model of ErbB signaling. (**a**) Heat map of ligand screen following subtraction of each cell lines median HSA control-based Akt signal and normalizing the signals within a cell line to the maximum ligand activation for that cell line. (**b**) Schematic depiction of the ErbB signaling network showing the receptors EGFR–ErbB4, BTC binding to EGFR and HRG binding to the ErbB3 receptor, receptor dimerization, dimer internalization and recycling, and interactions leading to activation of the PI3K-Akt cascade. The computational model is an interpretation of this schematic, using mass action kinetics. Because of the low expression observed *in vitro*, ErbB4 was omitted from the computational model. (**c**) The computational model was calibrated to a high-density experimental signaling data set. Phosphorylated-EGFR, HER2 and HER3− as well as p-Akt were measured in serum-starved ADRr ovarian cancer cells stimulated with HRG or BTC. The model was built in MATLAB SimBiology v2.1. A genetic algorithm was used to fit key parameters. Both experimental and simulated data are normalized to the largest signal for each target under either stimulus. (**d**) Sensitivity analysis of the ErbB model. The normalized time-integrated sensitivity of Akt phosphorylation to each non-zero species was determined by varying the amount of each non-zero species and simulating the time course of p-Akt in response to 1 nmol/l HRG or BTC, with the calibrated computational model. The normalized sensitivity integrated over the 2 h time course is shown, with species ranked according to their sensitivity during HRG stimulation. Figures 1b and c from Schoeberl *et al*.^1^ Reprinted with permission from AAAS.

**Figure 2:**
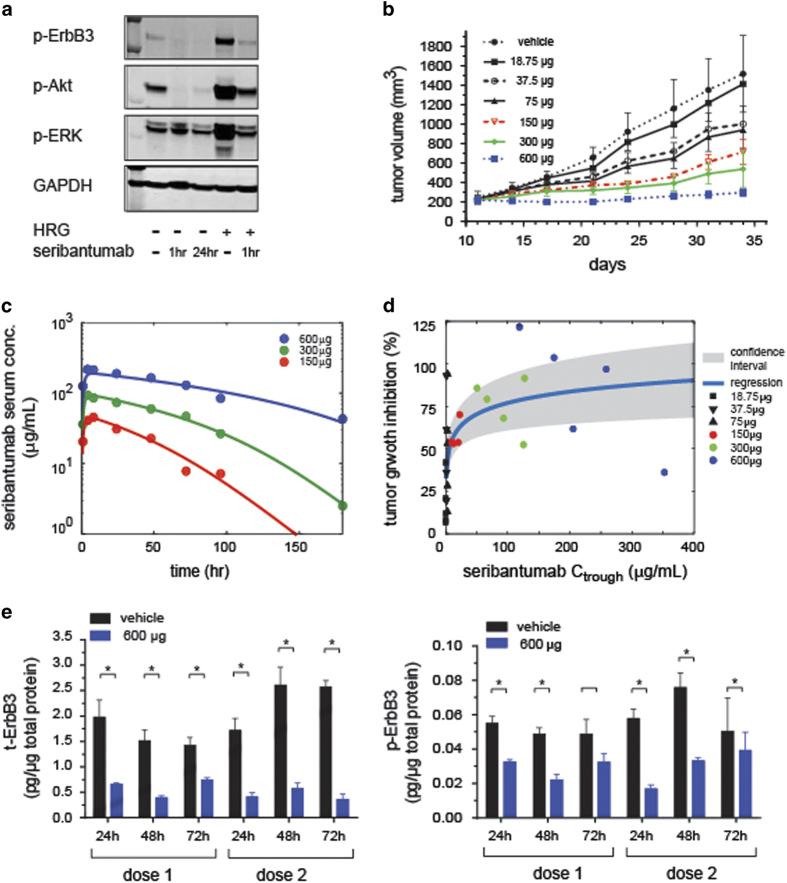
Single agent activity, pharmacokinetic and pharmacodynamic properties of seribantumab. (**a**) Seribantumab inhibits both basal and HRG-induced phosphorylation of ErbB3 and Akt in A549 cell lines. Serum-starved A549 cells were pre-treated with seribantumab (250 nmol/l) for 1 h or 24 h, followed by treatment with 10 nmol/l HRG for 10 min. (**b**) Tumor response in A549 xenografts following administration (μg/dose, q3d, intraperitoneal (i.p.)) of various seribantumab doses (*n*=5/group). Tumor growth was measured twice per week by calipers and plotted as mean±s.e.m. (**c**) Pharmacokinetic profile of seribantumab in serum obtained from A549 tumor bearing mice. Serum samples were collected at 1, 4, 8, 24, 48, 72, 96, and 168 h following single dose of seribantumab (*n*=3 mice/time point). A one-compartment pharmacokinetic model with first order absorption (lines) and non-linear clearance was used to fit the data set (dots) and estimate the pharmacokinetic parameters. (**d**) Relationship between tumor growth inhibition and serum trough levels following treatment with various doses of seribantumab (colored symbols represent experimental data and black symbols represent data at lower doses predicted using the PK model simulations). (**e**) Pharmacodynamic effects of seribantumab in A549 xenografts. Graph presents mean±s.d. of t-ErbB3 (left) and p-ErbB3 (right) levels as measured in A549 tumors following treatment with seribantumab (600 μg/dose, q3d, i.p). Tumors were harvested at 24, 48 and 72 h following either single dose or two doses of seribantumab (*n*=3 mice/time point). *P<0.05 versus control treated (by Wilcoxon rank-sum test).

**Figure 3:**
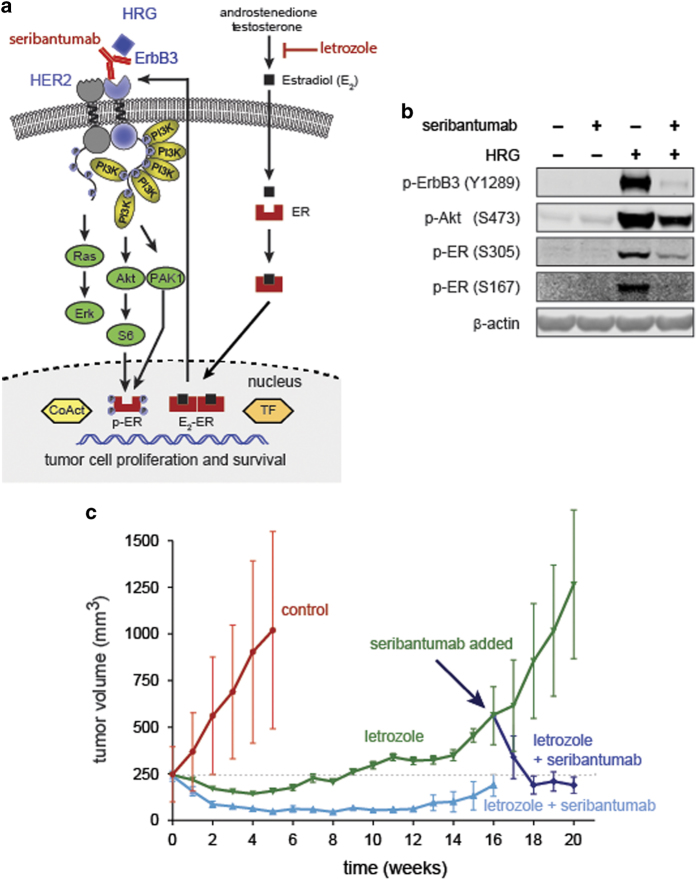
(**a**) Schematic illustration highlighting HRG-driven ErbB3 signaling as a mechanism mediating lack of responsiveness to endocrine therapy. (**b**) Seribantumab inhibits HRG-induced phosphorylation of ErbB3, Akt and ER in MCF-7Ca cells. Serum-starved MCF-7Ca cells were pre-treated with seribantumab (1 μmol/l) for 1 h, followed by treatment with 10 nmol/l HRG for 10 min. Cell lysates were analyzed by immunoblotting with antibodies for p-ErbB3 (Y1289), p-Akt (S473) and p-ER (S305 and S167). Anti-β-actin antibody was used as a loading control. (**c**) Seribantumab and letrozole co-treatment delays the onset of tumor tolerance to letrozole and restores sensitivity to letrozole in MCF-7Ca xenografts. MCF-7Ca xenograft tumors were generated in female, ovariectomized nude mice, which were randomized to receive vehicle (‘Control’; 0.3% HPC in 0.9% NaCl, twice weekly (Q2W), IP; 15 mice/group), seribantumab (750 μg/mouse, Q2W, IP; 15 mice/group), letrozole (10 μg/mouse/day×5 days/week (QD×5), subcutaneous injection (SQ); 60 mice/group), or letrozole in combination with seribantumab, dosed as indicated for the monotherapies (15 mice/group). Changes in mean tumor volume (±s.e.m.) were determined weekly by caliper measurement. Following the loss of sensitivity to letrozole (week 14), mice in the letrozole-only group were re-randomized into 15 mice/group to receive: letrozole alone; seribantumab alone; or a combination of letrozole and seribantumab.

**Figure 4:**
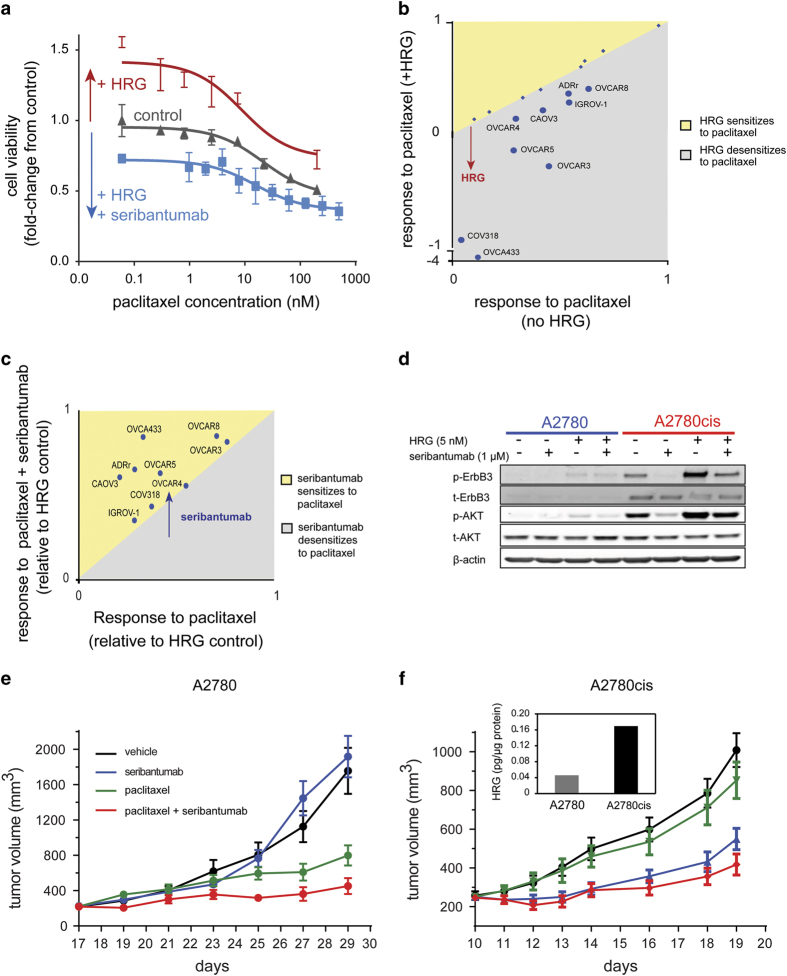
(**a**) The ovarian cancer cell line ADRr was treated with paclitaxel at increasing doses either alone (gray line), in the presence of 5 nmol/l HRG (red line) or in the presence of 5 nmol/l HRG and 1 μmol/l seribantumab (blue line). The graph illustrates the relative cell viability (normalized to media control) in a 96 h spheroid formation assay with CellTiter Glo as readout of viability. The arrows highlight the effect of HRG or HRG in combination with seribantumab on the response to paclitaxel. (**b**) A panel of ovarian cancer cell lines was screened using the same assay as in (**a**). The Area Under the Curve (AUC) fold-change relative to media control of all ovarian cancer cell lines screened, was plotted for paclitaxel in the absence or presence of HRG. Diamond shapes indicate cell lines that are non-responsive to HRG and the larger circles represent the HRG-responding cell lines. The gray region in the plot represents the area where HRG desensitizes cells to paclitaxel. (**c**) The AUCs of all HRG-responding cell lines screened was calculated for paclitaxel in the presence of HRG, with or without 1 μmol/l seribantumab. The yellow and gray represent the areas in which seribantumab sensitized versus desensitized cells to the drug, respectively. (**d**) Seribantumab inhibits both basal and HRG-induced p-ErbB3 and p-Akt in A2780cis. Serum-starved A2780 and A2780cis were pre-treated with seribantumab (1 μmol/l) for 24 h, followed by treatment with 10 nmol/l HRG for 10 min. A2780cis cells displays upregulation of basal p-ErbB3 and p-Akt compared with parental A2780 cell line. *In vivo* activity of seribantumab in A2780 (**e**) and A2780cis (**f**) ovarian cancer xenografts. Tumors were established subcutaneously (s.c.) in nu/nu mice. Following randomization, animals were treated with vehicle control (PBS), seribantumab (600 μg/dose, q3d, intraperitoneal (i.p.)), paclitaxel (40 mg/kg, q7d, i.p.) or combination of both drugs (*n*=8/group). Tumor volumes were calculated following caliper measurement and plotted as mean±s.e.m.

**Figure 5:**
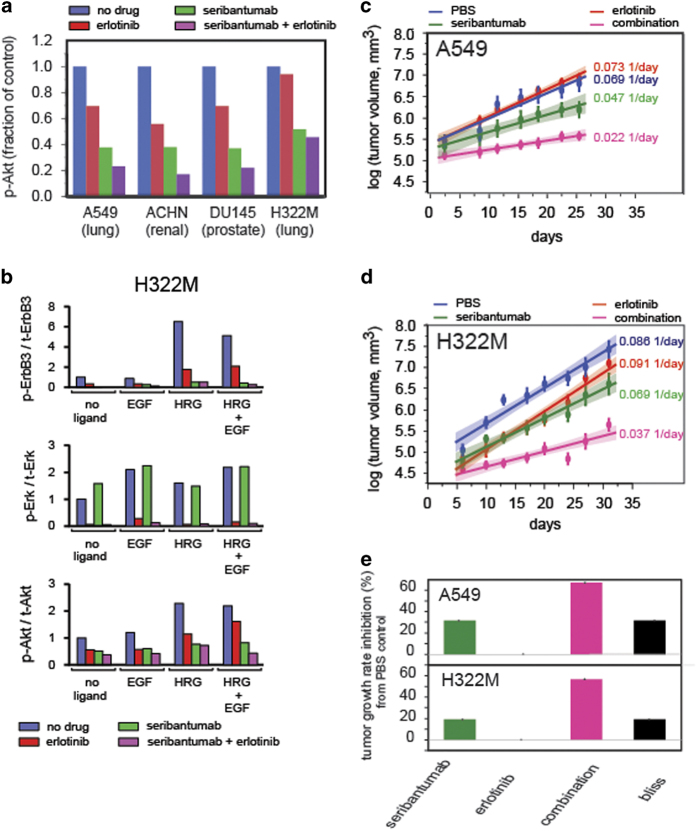
Dual Targeting of EGFR and ErbB3: (**a**) Mechanistic model to predict the response of seribantumab and erlotinib combination in A549, ACHN, DU145 and H322M cells. The initial conditions for the four cell lines (e.g., receptor levels) were set to those measured by qFACS under basal conditions (Supplementary Table S3).For each cell line the simulation was run for 30 min to allow the receptors to equilibrate. The inhibitors were then introduced for an additional 30 min, followed by virtual stimulation with HRG (1 nmol/l) and BTC (1 nmol/l) for 10 min to assess the effect of the inhibitors on Akt phosphorylation. Values are normalized to cells treated with HRG and BTC alone. (**b**) *In vitro* signal inhibition with the combination of seribantumab and erlotinib in H322M cells. Serum-starved H322M cells were pre-treated with either seribantumab (1 μmol/l), erlotinib (1μmol/l) or the combination for 30 min, followed by treatment with different ligands; HRG (10 nmol/l) alone, EGF (10 nmol/l) alone or both ligands for 1 h. Cell lysates were used for western blot analysis. *In vivo* activity of seribantumab in A549 (**c**) and H322M (**d**) xenografts. Subcutaneous tumors were established in nu/nu mice. Following randomization, animals were treated with vehicle control (PBS), seribantumab (300 μg/dose, q3d, intraperitoneal (i.p.)), erlotinib (25 mg/kg, q3d, oral gavage) or combination of both drugs (*n*=8/group). Tumor growth was measured twice per week by calipers and plotted as mean±s.e.m. and plotted on a log scale to assess the tumor growth kinetics for each treatment arm. (**e**) Tumor growth rate inhibition for A549 and the H322M xenograft model for the individual treatment arms and the combination compared with Bliss independence.

**Figure 6:**
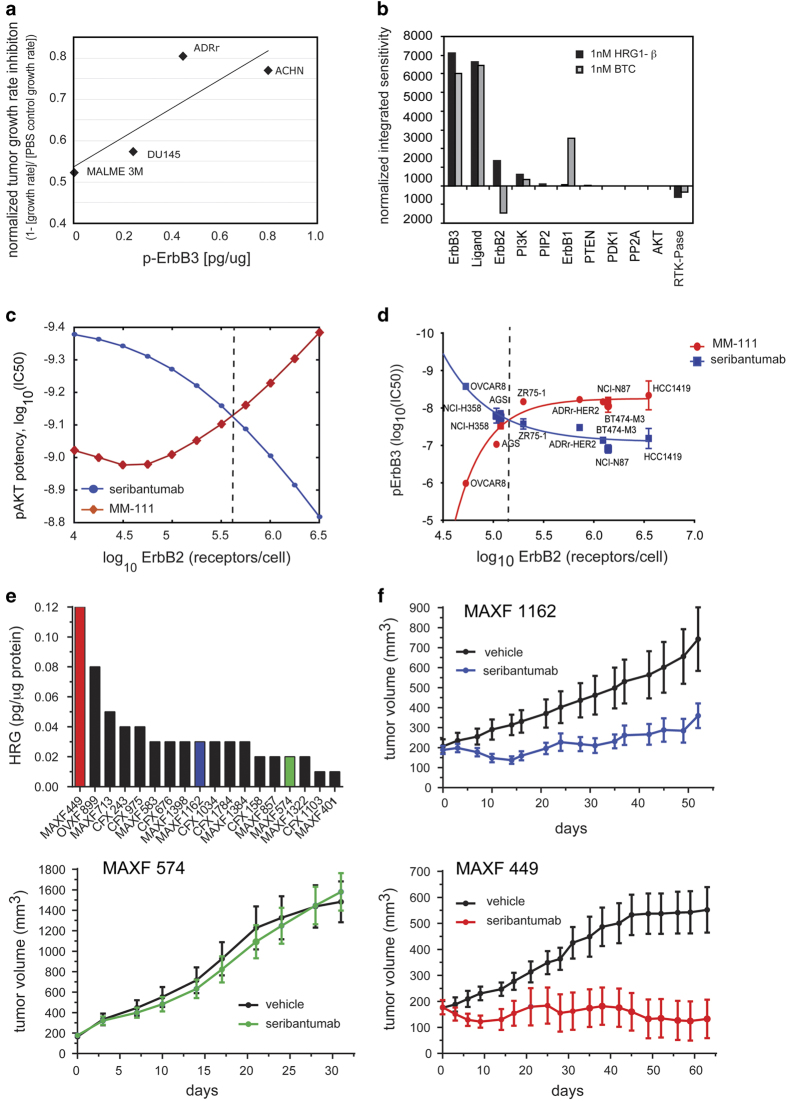
(**a**) Normalized tumor growth rate inhibition observed in MALME 3 M, DU145, ADRr and ACHN cell lines treated q3d with 300 μg/dose seribantumab plotted as a function of p-ErbB3 levels measured by ELISA in untreated tumors of about 200–300 mm^3^. (**b**) Sensitivity analysis of the ErbB model. The normalized time-integrated sensitivity of p-ErbB3 phosphorylation to each non-zero species was determined by varying the amount of each non-zero species and simulating the time course of p-ErbB3 in response to 1 nmol/l HRG or BTC, with the calibrated computational model. The normalized sensitivity integrated over the 2 h time course is shown, with species ranked according to their sensitivity during HRG stimulation. (**c**) Simulations results indicate that an anti-ErbB3 antibody (seribantumab, in blue) would be more potent in the HER2 low setting, while an ErbB3/HER2 bispecific molecule would be most effective in the HER2-high setting. In the simulated experiment, after 30 min incubation with a dose titration of MM-111 or MM-121 cells expressing different levels of HER2 were stimulated for 10 min with 1 nmol/l HRG. The p-Akt IC50 values derived from the simulations are plotted as a function of the HER2 expression levels. (**d**) Experimental validation of the simulated observation that the potency of MM-111 and seribantumab vary with the HER2 levels. The annotated cell lines were cultured in 4% serum, stimulated for 5 h with 5 nmol/l HRG, followed by drug treatment for another 5 h. Total ErbB3 and p-ErbB3 was measured using ELISA and IC50 curves fitted and plotted against the total ErB2 levels by qFACS. (**e**) HRG levels in patient-derived xenograft models. Tumor lysates were prepared from untreated tumors and HRG levels determined using ELISA method. (**f**) Single agent activity of seribantumab measured in select patient-derived xenografts; MAXF449, MAXF1162 and MAXF574. Tumors were established subcutaneously in nu/nu mice. Following randomization, animals were treated with vehicle control (PBS) or seribantumab (600 μg/dose, q3d, i.p.) Tumor growth was measured twice per week by calipers and plotted as mean±s.e.m. (*n*=8/group).

**Figure 7:**
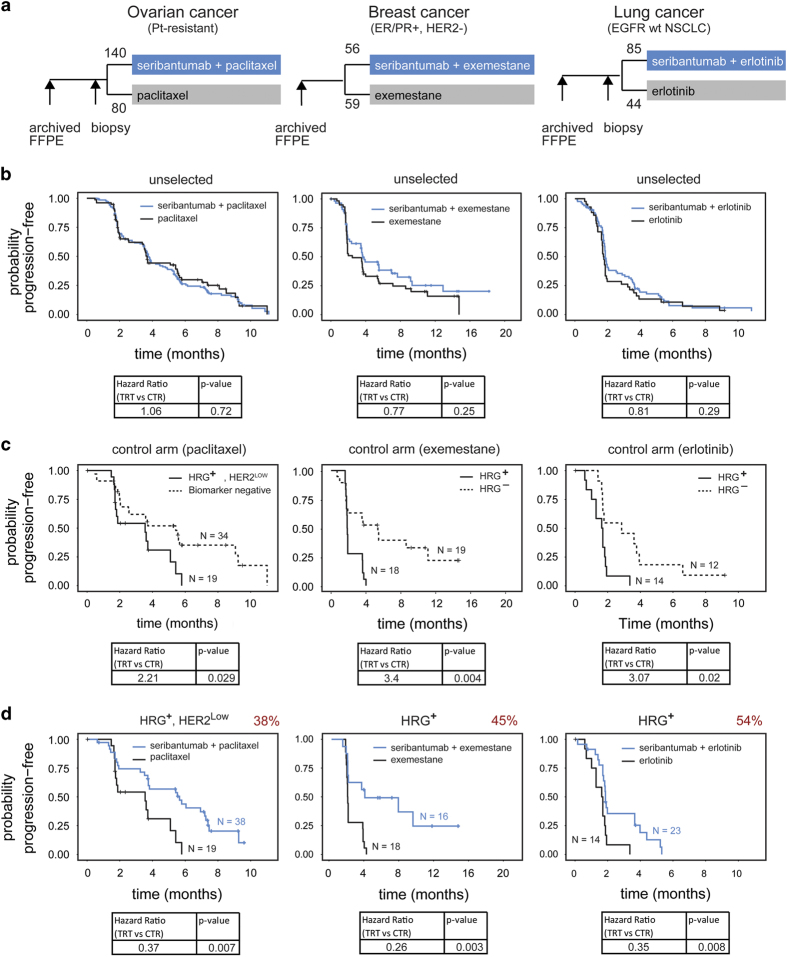
(**a**) Graphical description of the trial design of the three randomized Phase 2 trials in metastatic cancer: in combination with paclitaxel versus paclitaxel alone in platinum-resistant/refractory ovarian cancer; in combination with exemestane versus exemestane plus placebo in ER/PR+, HER2− breast cancer and in combination with erlotinib versus erlotinib alone in EGFR wild-type non-small cell lung cancer. (**b**) Kaplan-Meier plots of progression-free survival (PFS) in the unselected population across the three trials and the observed Hazard Ratios. (**c**) HRG appeared to be a prognostic marker of rapid progression on the control arm as indicated by the Kaplan-Meier plots of PFS of the control arm in the biomarker positive versus the biomarker negative population. (**d**) HRG+ patients appeared to derive benefit from seribantumab by comparing Kaplan–Meier PFS plots of the experimental arm with the control arm.

Here, we summarize a decade-long effort to apply the principles of Systems Biology to the discovery and development of a new investigational agent, seribantumab (MM-121; Merrimack). We will illustrate how systems-level investigations coupled with computational modeling were used to guide decision making, from target identification to therapeutic design (Figure 1), dose selection and single agent activity (Figure 2), to the identification of rational combinations (Figures 3, 4, 5) and finally to the preclinical identification of biomarkers (Figure 6) and clinical testing (Figure 7). The road began with a screen to identify the molecular pathways most critically involved in ligand-mediated cancer cell survival. These efforts highlighted the ErbB network, which was then modeled and dissected to identify the target—ErbB3—that is most critically involved in mediating pro-survival signaling. Computational models were used to assess how best to target ErbB3 and to define design criteria for the ensuing antibody, seribantumab. Modeling was further used to support dose selection and to identify candidate biomarkers for ErbB3 activation, and clinical trials designed to test and refine these biomarker hypotheses. The clinical development of seribantumab is not yet complete. To date, this agent has been tested in three randomized Phase 2 trials of metastatic cancer: in combination with paclitaxel versus paclitaxel alone in platinum-resistant/refractory ovarian cancer (NCT01447706); in combination with exemestane versus exemestane plus placebo in ER/PR+ HER2− breast cancer (NCT01151046); and in combination with erlotinib versus erlotinib alone in EGFR wild-type NSCLC (NCT00994123). Although seribantumab did not demonstrate significant clinical benefit in unselected patient populations, predefined biomarker analyses highlighted a subset of patients in all three settings that derived benefit from the addition of seribantumab to standard therapy. Consistent with both modeling and preclinical experiments, clinical benefit was observed in patients who’s tumors identified with a substantial subset of HRG expressing cancer cells detectable in their primary tumors or metastatic lesions, once again demonstrating the challenges associated with tumor cell heterogeneity. Seribantumab is currently being investigated in combination with chemotherapy versus chemotherapy alone in a Phase 2 study in NSCLC in a prospectively selected HRG-positive patient population (NCT02387216) following immunotherapy. Broader analysis of cancer patient outcomes across multiple indications suggests that the presence of HRG in tumors is emblematic of a patient population, that is characterized by inferior clinical outcomes with a demonstrated lack of efficacy to standard therapies. In this article, we highlight some of the clinical characteristics of patients displaying HRG-ErbB3 pathway activation, which we believe identifies a novel clinical phenotype characterized by the persistence of highly drug-tolerant HRG-positive cancer cells within a heterogeneous tumor that directly impacts clinical outcomes following anticancer therapy. Given our preclinical and clinical data to date, anti-ErbB3 therapies like seribantumab may have the potential to significantly enhance clinical activity of standard of care therapies by combating persistent drug-tolerant cancer cells via the inhibition of the HRG-ErbB3 pathway.

## Results

### Target identification and molecule design criteria

Although many cancer patients respond well to therapy, others do not respond at all and, even among those that initially respond, resistance inevitably arises. It had previously been shown that constitutive or inducible Akt phosphorylation promotes resistance to chemotherapy, targeted therapy (trastuzumab), and anti-hormonal therapy (tamoxifen).^18^ We reasoned that cancer cells could potentially evade all classes of therapy by eliciting or using signals in their extracellular environment to activate pro-survival signaling through Akt. Based on this hypothesis, we conducted a ‘Critical Network Identification’ screen to assess which of the myriad cytokines and growth factors known to activate Akt are the most potent across the NCI-60 panel of cell lines. The NCI-60 panel is a well-characterized collection of cell lines representing nine different tumor types.^19^ Focusing on the 54 cell lines in this panel that were derived from solid tumors, we exposed each cell line to saturating concentrations of 60 different growth factors or cytokines and, following 30 min of stimulation, measured the level of phosphorylated Akt at serine 167 (p-Akt (S473)) by ELISA (Figure 1a, Supplementary Table S1). Even though all 60 ligands had previously been reported to induce Akt phosphorylation, only five families of growth factors exhibited widespread activity across the cell line panel: the EGF ligand family; the heregulin (HRG) family; insulin-like growth factors 1 and 2 (IGF-1/2); hepatocyte growth factor (HGF); and the platelet-derived growth factors (PDGF family). Similar results were later observed by Niepel and colleagues in a broad panel of breast cancer cell lines.^12^

Given that both the EGF and HRG families of ligands induced Akt phosphorylation in most cell lines, we focused our computational modeling efforts on the ErbB signaling network. We started by defining the topology of the ErbB network based on our understanding of which ligands bind which receptors, how receptors homo- and heterodimerize, how receptors traffic, and which intracellular signaling pathways are activated. This information, as represented in cartoon form in Figure 1b, was captured in computational models comprising ordinary differential equations. There are four distinct ErbB receptors and 13 soluble and membrane-bound ligands that activate multiple downstream signaling pathways, including the mitogenic Ras-MAPK cascade and the pro-survival PI3K/Akt pathway.^20^ To better understand information flow through this network and to address specific scientific questions, we built two different models. The more extensive model captures Akt and Erk signaling induced by EGF and HRG,^21^ whereas the second, smaller model focuses specifically on activation of Akt by HRG and BTC.^1^ Both models capture homo− and heterodimerization among the ErbB receptors and were built to understand HRG, EGF, and BTC-induced signaling. BTC activates all four ErbB receptors, whereas EGF is more selective for EGFR and HRG is selective for ErbB3.^1^ Both models were constrained using all available kinetic parameters and then further trained using experimental data based on quantitative measurements of time-dependent signaling *in vitro*. Figure 1c shows a trained computational model, where kinetic parameters have been optimized to best match simulation results with experimental data.^1^ In this example, the simulated dose-time matrix for the ADRr ovarian cell line stimulated with either HRG or BTC (solid lines) closely matches the experimental data (circles). Capturing the complexity of ErbB signaling in a computational model enables new hypotheses to be formulated and tested quickly. For example, dynamic simulations, coupled with experimental data, revealed that when BTC binds to EGFR, it induces phosphorylation of ErbB3 to the same extent as HRG, but in a more transient manner. This observation prompted us to include in our model that EGFR, when bound to BTC, must heterodimerize with ErbB3.

Once the computational models had been trained and validated, we conducted an *in silico* sensitivity analysis to determine which proteins, when perturbed, have the most impact on Akt phosphorylation. In principle, a good drug target would be highly sensitive to perturbation, enabling a small effect on the target to translate into a profound effect on the cell. When we performed this analysis, ErbB3 was identified as the most sensitive node in the ErbB network, independent of which ligand was driving signaling (Figure 1d). This finding was further confirmed using the more comprehensive ErbB model.^21^ ErbB3 does not meet the conventional criteria of a drug target in cancer: it is generally expressed at low levels, is not gene amplified, is rarely mutated, and has very weak kinase activity.^22^ EGFR and HER2, which are considered validated drug targets, were also identified as sensitive targets for Akt phosphorylation, but their sensitivity is more dependent on the identity of the ligand driving pathway activation.

On the basis of these modeling insights, we defined specific design criteria for a therapeutic anti-ErbB3 monoclonal antibody. According to our models, the ideal antibody would have a sub-nanomolar monovalent binding affinity for ErbB3, would block HRG from binding to ErbB3, would inhibit BTC-induced ErbB3 phosphorylation via EGFR, and would trigger downregulation of ErbB3. We selected fully human antibodies to the extracellular domain of ErbB3 using phage display and screened candidate molecules for the one antibody, seribantumab, that met all of our design criteria.^1^ To avoid potential immune-mediated on-target toxicities, we elected to develop an antibody with an IgG2 Fc backbone, which does not elicit antibody-dependent cellular cytotoxicity. Subsequent to these initial efforts, several other companies also developed ErbB3-targeting monoclonal antibodies, many of which are currently in clinical development.^23^

### Single agent activity and dose selection

Figure 2 summarizes the single agent activity of seribantumab *in vitro* and *in vivo*. Seribantumab inhibits both basal and HRG-induced phosphorylation of ErbB3 after 1 h or 24 h of treatment in A549 lung carcinoma cells. This translates directly into inhibition of Akt phosphorylation and, to a lesser extent, Erk phosphorylation (Figure 2a). Similar results are observed across a variety of cell lines.^24^ Notably, inhibition of ErbB3 signaling by seribantumab *in vitro* translates into single agent anti-tumor growth activity in mouse xenografts. For example, treatment with seribantumab controls the growth of A549 tumors in a dose-dependent manner with maximal inhibition of tumor growth observed at a dose of 600 μg administered every 3 days (Figure 2b).

To inform a dosing strategy for the clinical administration of seribantumab, we modeled how inhibition of tumor growth varies with seribantumab serum concentration. Figure 2c shows the results of fitting mouse serum levels of seribantumab to a one-compartment pharmacokinetic model with first order absorption and non-linear clearance. Since the terminal half-life of seribantumab in mice increased with higher dose levels, non-linear saturable elimination process was used in the PK model. The clearance rate of seribantumab gets accelerated when the serum concentrations are lower than ~50 μg/ml (Figure 2c). As we were unable to measure seribantumab serum levels in the lower dose groups due to assay sensitivity, we simulated the serum levels for these doses. We then plotted the tumor growth rate inhibition as a function of seribantumab serum trough levels based on a q3d dosing schedule (Figure 2d). We found that serum levels of seribantumab above 50 μg/ml resulted in greater than 50% tumor growth inhibition. For the 600 μg q3d dose group of seribantumab, 40–125% inhibition of tumor growth was observed, and serum levels of seribantumab remained above 100 μg/ml. On the basis of these findings, we set a goal to maintain seribantumab trough levels above 100 μg/ml (667 nmol/l) in our clinical studies.

To determine if seribantumab is inhibiting tumor growth by the intended mechanism, we collected tumors from the dose-ranging mouse xenograft study and measured both total and phosphorylated levels of ErbB3 by ELISA. Figure 2e shows the impact of either one or two doses of seribantumab (600 μg/dose) on the total and phosphorylated levels of ErbB3 measured 24, 48, or 72 h after dosing the mice. Comparing pharmacodynamic effects following the first and second dose of seribantumab, we found that maximal inhibition and downregulation of ErbB3 is rapidly achieved (*P*<0.05 by Wilcoxon rank-sum test in all paired groups except for p-ErbB3 at 72 h), even after a single dose of the antibody.

### The cancer biology of ErbB3

Having engineered seribantumab to effectively inhibit ligand-mediated ErbB3 signaling, we turned our efforts to determining specific areas of cancer biology where ErbB3 blockade could potentially provide clinical benefit. The central hypothesis underlying seribantumab development is that ligand-driven signaling through ErbB3 serves as a widespread cancer cell survival pathway, rendering cancer cells tolerant to standard of care therapy, regardless of the class of therapy. Previously, three different ErbB3-mediated resistance mechanisms had been described: (i) increased levels of ErbB3 by enhanced *ERBB3* transcription; ii) increased HRG autocrine signaling; and (iii) ligand-independent activation of ErbB3 by other RTKs such as HER2 and Met.^25–27^ To test our clinical hypothesis broadly, we focused on the three major classes of systemic cancer therapy: endocrine therapy, chemotherapy, and targeted therapy. In each case, we sought to understand if ErbB3 signaling is a potential mechanism promoting drug tolerance and asked if ErbB3 inhibition enables tumor cells to respond to these agents. These three areas are addressed in the following three sections and formed the basis of the Phase 2 clinical development program for seribantumab.

### HRG/ErbB3 bypasses anti-hormonal therapy in breast cancer

Approximately 80% of breast cancers express estrogen receptor (ER), progesterone receptor (PR), or both, and are therefore considered hormone receptor-positive (HR+). Patients with HR+ breast cancer often respond favorably to drugs such as exemestane and letrozole, which inhibit hormone production by blocking the enzyme aromatase, or to drugs such as tamoxifen and fulvestrant, which act directly on ER to block estradiol-dependent signaling. Collectively, this class of drugs is referred to as anti-hormonal or endocrine therapy. As with other classes of therapy, many patients with advanced HR+ breast cancer encounter *de novo* or acquired resistance and require more aggressive treatment options such as chemotherapy.

One prominent way in which resistance to endocrine therapy arises is through activation of alternative signaling pathways that are either intrinsically present or drug-induced. Notably, ER can be activated not only by estradiol, a derivative of estrogen, but by a variety of growth factors that act in an estrogen-independent manner.^28^ As shown in Figure 3a, HRG-induced ErbB3 signaling can lead to activation of ER through phosphorylation on serine 167 (Ser167) via the PI3K/Akt pathway^29^ and through phosphorylation on serine (Ser305) via the PI3K/p21-activated kinase 1 (Pak1) pathway.^30^ In addition, it has been shown that anti-hormonal therapies like fulvestrant rapidly induce *ERBB3* expression, further sensitizing cells to HRG.^31,32^ Indeed, the rapid induction of *ERBB3* expression may constitute a key mechanism of acquired resistance to endocrine therapy.

In our own hands, *in vitro* stimulation of MCF-7Ca ER+ breast cancer cells with HRG led to rapid phosphorylation of ErbB3 and Akt, as well as phosphorylation of ER on Ser167 and Ser305 (Figure 3b). Pre-incubation with 1 μmol/l seribantumab, on the other hand, resulted in complete inhibition of ErbB3 phosphorylation, partial inhibition of Akt phosphorylation, and reduction or complete inhibition of ER phosphorylation on Ser305 and Ser167, respectively.^33^ On the basis of these data, we sought to determine if ErbB3 signaling is active in MCF-7Ca-based xenograft models, and if blocking this pathway affects tumor growth. MCF-7Ca cells, which are engineered to express aromatase, were implanted subcutaneously in ovariectomized mice. In this model, tumors initially respond to the aromatase inhibitor letrozole, but spontaneously become letrozole tolerant and continue to grow despite prolonged exposure. As anticipated, treatment with letrozole initially led to tumor stasis, but was followed by persistent tumor progression around week 14 (Figure 3c). In contrast, mice that were co-treated with seribantumab and letrozole continued to show tumor stasis at week 14, indicating that seribantumab can delay the onset of tumor drug tolerance. Once tumor progression emerged in the cohort of mice treated with only letrozole, the mice were re-randomized and treated either with letrozole alone or the combination of letrozole and seribantumab. In the cohort of mice receiving combination therapy, the tumors that had progressive tumor growth rapidly regressed, returning to the size of tumors in the cohort at the beginning of treatment.^33^ This suggests that the persistence of HRG-positive cancer cells observed in tumors from patients with advanced breast cancer will directly impact clinical outcomes of patients receiving endocrine therapies. Co-administration of seribantumab and the endocrine therapy such as AIs is warranted to effectively combat the tumor holistically. Seribantumab would not be expected to act as a single agent since it essentially converts HRG-positive cancer cells into phenotypically HRG-negative cancer cells and as such allows effective targeting of all cancer cells through the combination drug.

### HRG/ErbB3 blunts the cytotoxic activity of chemotherapy in ovarian cancer

In breast cancer, there is a direct mechanistic reason why HRG overcomes endocrine therapy in drug-tolerant HRG-positive ‘persister’ cells: ErbB3 signaling activates ER in an estrogen-independent manner. In the case of cytotoxic chemotherapy, however, it appears that ErbB3 sends a general, pro-survival signal to the cell, abrogating the cytotoxic effects of a broad range of chemotherapeutic agents. To illustrate, when ADRr ovarian cancer cells are treated with increasing doses of paclitaxel (a microtubule-stabilizing drug), cell viability is inhibited in a dose-dependent manner (Figure 4a). When the same experiment is performed in the presence of 1 nmol/l HRG, the dose-response curve is shifted toward increased cell viability, even at relatively high concentrations of paclitaxel (100 nmol/l–1 μmol/l). In the presence of seribantumab, however, HRG-mediated insensitivity is alleviated. In fact, the dose-response curve to paclitaxel in the presence of HRG and seribantumab falls below the control curve, suggesting the presence of an autocrine HRG loop in these cells. By plotting the area under the curve (AUC) for paclitaxel in the presence or absence of HRG for multiple ovarian cancer cell lines, we found that HRG renders ~50% of these cell lines insensitive to paclitaxel (Figure 4b). When the HRG-responding cell lines were treated with HRG and seribantumab in combination with paclitaxel, all of the cell lines were re-sensitized to paclitaxel basically converting them back to HRG-negative cells (Figure 4c).

Although these experiments show that HRG/ErbB3 signaling has the ability to render ovarian cancer cells insensitive to paclitaxel, the question remains whether or not cancer cells invoke this mechanism to adapt to the stress of cytotoxic therapy. Behrens and colleagues previously described a chemo-resistant cell line, A2780cis, that they generated by chronic exposure of the drug-sensitive ovarian cell line A2780 to increasing concentrations of cisplatin.^34^ Notably, the resistant cell line has higher levels of total ErbB3 than the parental line, and higher basal activation of both ErbB3 and Akt (Figure 4d). It is also much more responsive to stimulation with HRG. Treatment with seribantumab inhibited both basal and HRG-induced activation of ErbB3 and Akt in the chemo-resistant cell line (Figure 4d). When grown as subcutaneous xenografts in mice, the parental line (A2780) responded to paclitaxel, but was unresponsive to seribantumab (Figure 4e). In contrast, tumors derived from the resistant cell line (A2780cis) did not respond to either cisplatin (not shown) or paclitaxel (Figure 4f), but were clearly responsive to seribantumab (Figure 4f). Consistent with this observation, HRG levels were elevated in A2780cis tumors relative to A2780 tumors (Figure 4f inset), supporting the notion that insensitivity to chemotherapy is ErbB3-mediated. Importantly, this is also observed clinically. In late-stage ovarian cancer patients, approximately 30% of primary tumor cells derived from malignant ascites fluid show constitutive active p-ErbB3, which is inhibited by seribantumab.^35^

### Dual targeting of EGFR and ErbB3 is synergistic in non-small-cell lung cancer

In addition to empirically discovering drugs like paclitaxel that combine well with seribantumab, we also used our computational models to predict which targeted agents would mechanistically synergize with seribantumab to inhibit p-Akt.^36^ On the basis of dynamic simulations, the combination of an anti-EGFR inhibitor with seribantumab emerged as the most potent way to inhibit Erk and Akt activation in the presence of EGF and HRG. Erlotinib was chosen as a representative EGFR inhibitor, based on its clinical use in NSCLC. Simulations were performed at 1 μmol/l of erlotinib and 1 nmol/l of seribantumab, in the presence of 1 nmol/l HRG and 1 nmol/l BTC across four different cell lines (Figure 5a). The ligands and ligand concentrations were chosen to mimic autocrine signaling. We chose suboptimal drug doses in order to observe the combination effect. Erlotinib was implemented into the computational model similar to lapatinib, as previously reported^1^ and included in the updated model code in Supplementary Table S4. Additional biochemical reactions without impact on the results of the original model simulations were included in the model to enable the simulation of drug combinations in the presence of HRG and BTC as described in the Materials and Methods and model code (Supplementary Table S4). We tested our predictions experimentally by measuring p-ErbB3, p-Akt, and p-Erk in response to ligand stimulation and drug treatment as shown for the H322M cell line in Figure 5b. Whereas seribantumab was effective at inhibiting HRG-induced p-ErbB3 and p-Akt and erlotinib was effective at inhibiting EGF-induced p-Erk, only the combination of both drugs shut down both pathways in the presence of HRG and EGF, which is qualitatively in agreement with the simulation results.

Dual targeting of EGFR and ErbB3 most effectively blocked p-Erk and p-Akt in the presence of EGFR ligands and HRG, which translated into synergistic inhibition of tumor growth in A549 (Figure 5c) and H322 (Figure 5d). To better compare the effects of the different therapies, the tumor growth curves were fitted to exponential functions for each mouse within each treatment cohort (Figure 5c and Figure 5d). By comparing the experimentally observed inhibition of tumor growth with the predicted tumor growth rate inhibition using a Bliss additivity model,^37^ we found that the combination of seribantumab and erlotinib is synergistic in the A549 and H322M xenograft models (Figure 5e). These results are consistent with those observed with other ErbB3 inhibitors. For example, AV-203, an anti-ErbB3 antibody developed by Aveo Oncology, was shown to reverse ErbB3-induced tolerance to targeted therapies like erlotinib and lapatinib *in vitro*.^38^

### Preclinical biomarker hypotheses based on computational modeling

Because seribantumab is designed to block ErbB3 signaling, we only expect it to be effective in cancers in which a substantial proportion of cancer cells with active signaling persists within a heterogeneous tumor. In the early stages of drug development, however, it is not always obvious how best to diagnose pathway activation. This is another circumstance in which the tools of Systems Biology can prove useful. In preclinical experiments, we found that the degree of tumor growth rate inhibition by seribantumab correlated linearly with basal levels of p-ErbB3 measured in tumors of mouse xenograft models (Figure 6a). It is challenging, however, to measure p-ErbB3 levels in tumor biopsies because ErbB3 is rapidly dephosphorylated once the tumor sample is removed from the patient (i.e., in ischemic tissue).^39^ We, therefore, sought to identify stable biomarkers that could serve as surrogates for p-ErbB3. Using our computational model we ran a sensitivity analysis to identify biomarkers whose levels most profoundly affect the degree to which ErbB3 is phosphorylated: the ligands HRG and BTC; and the receptors ErbB3, HER2, and EGFR (Figure 6b). To challenge our model predictions, we also investigated the potential role of genetic biomarkers in modulating seribantumab activity. As described in detail by Yarar and colleagues., for example, we found that activating mutations in PIK3CA did not substantially affect response to seribantumab;^40^ therefore we concentrated on our five preclinical biomarkers and developed clinically applicable assays that could be used to quantify these biomarkers at either the protein or mRNA level: fluorescence-based quantitative IHC assays for the receptors (protein level); RNA-in situ hybridization assays for the ligands (mRNA level); and reverse transcriptase–quantitative PCR assays for all five biomarkers (mRNA level).

For the ligands HRG and BTC, it is intuitive that higher levels of these biomarkers would lead to higher levels of ErbB3 signaling and hence increased need for an ErbB3 inhibitor. Similarly, we would expect that higher levels of ErbB3, HER2, and EGFR would all correlate with increased signaling and hence increased benefit from seribantumab. We were, therefore, surprised when model simulations predicted the opposite for HER2. By modulating the levels of HER2 *in silico*, we found that the potency of p-Akt inhibition by seribantumab decreases as HER2 levels increase (Figure 6c). This prompted us to develop, in parallel, a second ErbB3-targeted agent, MM-111, that co-targets HER2 and ErbB3.^41^ This bispecific antibody, which comprises two scFv fragments connected by a human serum albumin linker, is designed to dock onto HER2 receptors on HER2-positive tumor cells and potently inhibit HRG/ErbB3 based on high avidity binding. Model simulations showed that, in contrast to seribantumab, MM-111 potency increases with increasing HER2 levels (Figure 6c). The biochemical equations describing the mechanism of action of MM-111 are included in the updated model code in Supplementary Table S4. These model simulations were tested experimentally by measuring the IC50 for p-ErbB3 inhibition of seribantumab and MM-111 in a variety of cell lines exhibiting a range of HER2 levels. As predicted, seribantumab was most potent in cells with low HER2 levels (<~200,000 receptors/cell), whereas MM-111 was most potent at inhibiting p-ErbB3 in cell lines with high HER2 levels (>~200,000 receptors/cell; Figure 6d). To test these insights, we measured the levels of all five biomarkers in 18 patient-derived xenograft models. We selected three models that expressed moderate-to-high levels of ErbB3, low levels of HER2, and a range of HRG levels (low, medium, and high; Figure 6e). As predicted, seribantumab had the greatest impact on the MAXF449 model, which had the highest HRG levels (Figure 6f). Moderate activity was observed with the MAXF1162 model, which had intermediate levels of HRG, and no activity was observed with the MAXF574 model, which had near undetectable levels of HRG. We also built a support vector machine (SVM) predictor of seribantumab activity based on the levels of these five biomarkers.^24^ The predictor was trained using data from eight cell line-derived xenograft models, which were classified as either responders to seribantumab (>50% tumor growth inhibition) or non-responders (<50% tumor growth inhibition). Using an independent test set of 12 additional models, the predictor accurately classified all 12 models. Notably, the biomarker providing the most predictive information in this classifier was HRG.

### Clinical investigation of seribantumab in late-stage, metastatic cancers

Having established that ligand-driven ErbB3 signaling either blunts or circumvents response to endocrine therapy, chemotherapy, and targeted therapy, and having defined a set of potential biomarkers of seribantumab activity, we next sought to test these hypotheses clinically. Seribantumab was first evaluated for safety in several Phase 1 trials, both as a single agent and in combination with a range of standard therapeutic drugs. In a first-in-human Phase 1 trial (NCT00734305), seribantumab did not induce partial or complete responses as a single agent, but appeared to induce prolonged stable disease in a subset of patients. It was generally well tolerated and combined safely with a variety of agents, including anti-hormonal therapies (exemestane; NCT01151046), targeted therapies (erlotinib, cetuximab, XL147; NCT00994123, NCT01451632, NCT01436565), and chemotherapies (paclitaxel, irinotecan, gemcitabine, carboplatin, pemetrexed, and cabazitaxel; NCT01209195, NCT01451632, and NCT01447225).^42–44^

Next, to determine if seribantumab could prolong progression-free survival, it was evaluated in three randomized Phase 2 trials in metastatic cancer (Figure 7a): in combination with paclitaxel versus paclitaxel alone in platinum-resistant/refractory ovarian cancer (NCT01447706);^45^ in combination with exemestane versus exemestane plus placebo in ER/PR+, HER2− breast cancer (NCT01151046);^46^ and in combination with erlotinib versus erlotinib alone in EGFR wild-type non-small-cell lung cancer (NSCLC; NCT00994123).^47^ Because it was not known which of the five biomarkers are sufficient to identify patients that would benefit from seribantumab, and at what levels, all three trials were designed to enroll ‘unselected’ patients, regardless of biomarker status, and then answer this question retrospectively. It was also not known how these biomarkers change as a function of treatment history or disease progression. We therefore collected archived tissue blocks if available which are typically acquired from a patient by surgical resection when they are initially diagnosed with cancer, as well as pre-treatment core-needle biopsies, which reflect the patient’s current disease state as indicated in Figure 7a. We then measured the five pre-specified biomarkers in each sample using the assays detailed above.^48^ Biomarkers were initially evaluated by fitting to a Cox proportional hazard model of biomarker-by-treatment interaction. Four biomarkers (HRG, ErbB3, BTC and HER2) and their associated assays were prioritized for further analysis and were directionally consistent with preclinical predictions and relate directly to the mechanism-of-action of seribantumab. Biomarkers showing a treatment interaction (*P*<0.4) were subsequently evaluated using two-variable models and thresholds were chosen based on local HR scans.

Although the three trials were run in parallel, the first to complete was the ovarian cancer trial (NCT01447706; Figure 7a). In this study, patients with advanced ovarian cancer, either resistant or refractory to platinum agents, were randomized 2:1 to receive seribantumab in combination with paclitaxel, or paclitaxel alone. The primary endpoint of the study was to determine if seribantumab, when added to paclitaxel, extended progression-free survival (PFS) relative to paclitaxel alone. Secondary objectives included assessing the effect of the five pre-specified biomarkers on PFS, as well as determining the effect of seribantumab on overall survival (OS) and objective response rate (ORR). In this study, the hazard ratio (HR) in the unselected patient population was 1.06 (95% CI: 0.76–1.48 stratified log-rank test *P*=0.719). Thus, there was no evidence that seribantumab extended PFS in the unselected patient population (Figure 7b). When the biomarker data were analyzed, however, three of the five biomarkers appeared to be predictive of benefit from seribantumab. Increasing levels of HRG and ErbB3 both correlated with decreasing (i.e., more favorable) hazard ratios, whereas decreasing levels of HER2 correlated with decreasing hazard ratios. All three of these effects were directionally consistent with preclinical predictions.

When pairs of biomarkers were tested, the best two-biomarker model comprised the combination of HRG and HER2 (i.e., these two biomarkers provided orthogonal information). A ‘biomarker-positive’ (BM+) subpopulation was therefore defined as patients having detectable levels of HRG (HRG+) and low levels of HER2 (HER2<126,000 receptors/cell). This two-variable biomarker appeared both prognostic of poor outcome for patients receiving paclitaxel alone and predictive of benefit from seribantumab in combination with paclitaxel. Focusing on the control arm patients (i.e., those receiving paclitaxel alone), the BM+ patients progressed rapidly relative to the BM− patients (hazards ratio (HR)=2.21; 95% confidence interval (CI): 1.08–4.51; *P*=0.029; Figure 7c). This is consistent with the hypothesis that HRG-driven ErbB3 signaling mediates insensitivity to paclitaxel. Comparing experimental and control arms, the biomarkers also appeared predictive of benefit from seribantumab: in the BM+ subpopulation, the PFS HR was 0.37 (95% CI: 0.18–0.76; *P*=0.007; Figure 7d).

The second study to complete was the breast cancer trial (NCT01151046; Figure 7a), in which patients with hormone receptor-positive, HER2-negative metastatic breast cancer were randomized 1:1 to receive either exemestane in combination with seribantumab, or exemestane in combination with placebo. As with the ovarian cancer study, the trial did not formally meet its primary endpoint of extending PFS in the unselected patient population, although in this case a trend was observed toward increased PFS in the experimental arm relative to the control arm (HR=0.772; 95% CI: 0.496–1.201; *P*=0.249; Figure 7b). Importantly, when the biomarker data were analyzed, HRG once again appeared both prognostic of poor outcome and predictive of benefit from seribantumab. On the control arm, the HRG+ patients progressed rapidly relative to the HRG− patients (HR=3.4; 95% CI: 1.48–7.85; *P*=0.004; Figure 7c). Similarly, comparing the experimental arm with the control arm, HRG+ patients appeared to derive benefit from seribantumab (PFS HR=0.26; 95% CI=0.11–0.63; *P*=0.003; Figure 7d). In this trial, it was deemed unnecessary to measure HER2. The trial was performed in HER2− breast cancer patients and the overwhelming majority of patients had HER2 levels that fell below the previously determined threshold of 126,000 receptors/cell.

Finally, the third study to complete was the lung cancer trial (NCT00994123; Figure 7a), in which patients with EGFR wild-type NSCLC were randomized 2:1 to receive seribantumab in combination with erlotinib at a dose of 100 mg/day, or erlotinib alone at 150 mg/day. The dose of erlotinib in the experimental arm was lower than in the control arm due to tolerability of the combination therapy. As with the other two studies, seribantumab did not significantly extend PFS in unselected patients (HR=0.81; 95% CI: 0.55–1.20; *P*=0.290; Figure 7b). Once again, however, HRG was prognostic of rapid progression on the control arm (HR=3.07; 95% CI: 1.19–7.91; *P*=0.0.020; Figure 7c) and predictive of benefit from seribantumab (HR=0.35; 95% CI: 0.16–0.76; *P*=0.008; Figure 7d). HER2 is rarely overexpressed in NSCLC patients and so was deemed unnecessary to measure.

Comparing data across the three clinical studies, the most predictive biomarker was HRG mRNA. In breast cancer, measurements were performed in archived tissue (pre-treatment biopsies were not collected), whereas in lung cancer, measurements were performed in pre-treatment biopsies (archived tissue was not available for most patients). Interestingly, we were able to collect both archived tissue and pre-treatment biopsies for most patients on the ovarian cancer trial.^49^ In this setting, we found that HRG mRNA was both prognostic and predictive, regardless of at which time point it was measured. We found, however, that HRG status changed during the course of disease progression. For those patients that tested HRG+ in their archived tissue, 78% remained HRG+ in their pre-treatment biopsies. In contrast, ~50% of patients that were HRG− in their archived samples tested HRG+ in their pre-treatment biopsies. This suggests that HRG-positive cells are further selected as patients go through various lines of therapy.

In addition to HRG mRNA, HER2 was also observed to be an important biomarker in ovarian cancer. It is also likely to be important in breast and lung cancer, but it was not necessary to measure as a biomarker as it is naturally low in lung cancer and already selected to be low in ‘HER2-‘breast cancer. Interestingly, the level of HER2 in ovarian cancer below which seribantumab provides benefit (~126,000 receptors/cell) closely matches the level predicted by computational modeling and preclinical experiments in which seribantumab potently inhibits ErbB3 phosphorylation (Figures 6c and d). Finally, consistent with our preclinical studies, we did not find any evidence clinically that PIK3CA mutations preclude seribantumab activity. On the basis of the collective results from these Phase 2 studies, along with the evolving treatment landscape in lung cancer, seribantumab is currently being evaluated in combination with chemotherapy in a randomized Phase 2 study in metastatic NSCLC in which patients are prospectively selected based on HRG tumor cell expression (NCT02387216).

## Discussion

With the advent of large-scale genomics efforts, cancer has been increasingly subdivided and categorized based on genetic abnormalities. Many of these mutations or gene amplifications are directly targetable, and with our current tools we are now frequently successful at identifying small molecules or therapeutic antibodies that inhibit their corresponding proteins with sufficient selectivity and potency. Although additional cancer-related genes no doubt remain to be identified, the fundamental challenge in cancer therapy is no longer finding new targets and drugging them effectively. Instead, it is understanding how cancers interact with their environment and rapidly adapt to avoid therapeutic intervention or attack from the immune system. To make a dramatic impact on patient survival, we need to learn: (1) how to match drugs with the specific disease state of the patient through precision diagnostics; (2) how to anticipate and avoid drug tolerance and immune system evasion through rational drug combinations and drug sequencing; and (3) how to optimize drug delivery and pharmacology to maximize therapeutic index.

One of the frustrations of cancer drug development is that targeted therapies are often less effective in the clinic than anticipated. Often, this is because we do not understand well enough how inhibiting a specific protein will impact the network in which that protein resides. This shortcoming is further compounded by genetic and epigenetic variability between patients that often dilutes the overall effect of a drug for reasons that are not well understood. Such heterogeneous response rates and lack of response durability suggests the existence of a multiple cancer phenotypes that are characterized by both a lack of response and/or loss of initial responsiveness. This further exemplifies the rather fluid evolutionary nature of many cancers and the significant clinical challenge that arises due to the existence of a patient population with heterogeneous tumors that are difficult to treat. This inherent complexity in cancer necessitates the development of computational models, whether at a single biological level, such as cell signaling, or at multiple levels, connecting molecular events to cell–cell communication to whole-body pharmacology.^50^

As a first step in applying computational modeling to the process of drug discovery and development, we used mechanistic modeling to identify ErbB3 as a novel target in the ErbB network. Although there are many druggable targets in this network, ErbB3 was highlighted as the most sensitive node with respect to Akt inhibition. Sensitivity is an important network characteristic in drug development, as it indicates which node, when perturbed to a small extent, has a large impact on the desired outcome. In this case, the desired outcome was to inhibit cancer cell survival, using p-Akt as a surrogate. We found that small decreases in either the total levels of ErbB3 or its activation by ligand would translate into a large impact on pro-survival signaling.

Beyond identifying ErbB3 as a target, modeling and simulation were used to design seribantumab. In the field of engineering, modeling has an integral role in defining design criteria. The same principles were applied to the design of seribantumab. Simulations were performed to determine the best way to inhibit ErbB3. In principle, ErbB3 inhibition can be accomplished in a variety of ways: by designing a ligand trap, which targets HRG; by designing a ligand blocker, which prevents ErbB3 from undergoing the conformational change necessary for receptor activation; by designing a dimerization blocker, which prevents ErbB3 from pairing with another ErbB receptor; or by designing a downregulator, which induces internalization and degradation of ErbB3. Simulations led us to design an antibody with a dual mechanism of action: one that blocks ligand binding and also induces receptor downregulation. During this process, simulations also highlighted how increasing levels of HER2 diminish seribantumab potency, leading us to design a second drug, MM-111, that inhibits HRG-driven ErbB3 signaling in HER2-high tumors.

Finally, modeling and simulation played a critical role in identifying potential biomarkers for seribantumab. Our models highlighted five biomarkers: all five biomarkers are sensitive nodes in the network with respect to ErbB3 activation (phosphorylation of ErbB3). Four biomarkers (HRG, ErbB3, BTC, and HER2) and their associated assays were prioritized for further analysis and were directionally consistent with preclinical predictions and relate directly to the mechanism-of-action of seribantumab.

Biomarkers showing a treatment interaction (*P*<0.4) were subsequently evaluated using two-variable models and thresholds were chosen based on local hazard ratio (HR) scans. Based on the trade-off between hazard ratio and prevalence, HRG and HER2 were identified as the most favorable pair of predictive biomarkers. In ovarian cancer, benefit from seribantumab was restricted to the patients with low levels of HER2 (<126,000 receptors/cell), whereas in the NSCLC and hormone receptor-positive and HER2-negative breast cancer study all tumor biopsies obtained tested below these HER2 levels. This threshold, observed clinically in the ovarian cancer study as the level below which the HR favored seribantumab (HR<1.0), closely matched results from model simulations predicting seribantumab potency decreases when HER2 levels rise above ~200,000 receptors/cell. The direct translation of this modeling result to the clinical setting shows that, even though preclinical results often do not translate from bench to bedside, mechanism-based predictions are more likely to bridge this gap.

One of the key findings from the Phase 2 studies of seribantumab is that HRG mRNA appears to be a clinically relevant biomarker for insensitivity to therapy identifying a significant subset of patients burdened by the presence of a substantial subset of HRG-positive cancer cells within their tumors potentially directly impacting clinical outcomes on standard therapies. Perhaps the best example is that of HER2, which, prior to the advent of trastuzumab, was indicative of breast cancer patients with particularly poor outcomes. HER2-positive breast cancer patients represented a molecularly defined subset of breast cancer that derived little benefit from chemotherapy. With the advent of trastuzumab, however, these patients now have a molecularly targeted therapy that dramatically improves patient outcomes. Similarly, NSCLC patients with activating mutations in EGFR are now routinely treated with erlotinib or other EGFR tyrosine kinase inhibitors, and testing for these mutations has become standard-of-care.^51^ In the clinic, we found that persistence of HRG mRNA expressing cancer cells is prognostic of poor response to standard therapy. In all three Phase 2 studies, HRG-positive patients’ tumors on the control arm progressed more rapidly than HRG-negative patients’ tumors, with hazard ratios in excess of 2.0 (Figure 7c). A similar result was obtained in the HERALD study of patritumab, another ErbB3-targeted monoclonal antibody developed by Daiichi Sankyo.^52^ In this Phase 1b/2 study, patritumab in combination with erlotinib was compared with patritumab plus placebo in EGFR inhibitor-naïve advanced NSCLC. A retrospective analysis of HRG mRNA levels, as assessed by reverse transcriptase–quantitative PCR in tumor tissue, showed that patients with HRG mRNA levels above the median progressed more rapidly on the control arm than patients with levels below the median.

These findings are also consistent with several epidemiological studies. Shames and colleagues analyzed HRG and ErbB3 mRNA levels in more than 750 tumors of diverse origin, including over 150 primary and recurrent tissue samples from patients with squamous cell carcinoma of the head and neck.^53^ They found that high HRG expression is associated with activation of ErbB3 (as assessed by ErbB3 phosphorylation) and that HRG expression was significantly higher in recurrent specimens compared with patient-matched and unmatched therapy-naïve specimens. These findings suggest that HRG expression may be both predictive of response to ErbB3 inhibitors and prognostic for cancer recurrence. Similarly, Qian and colleagues assessed the prognostic value of HRG mRNA and ErbB receptor protein levels in 96 patients with oropharyngeal squamous cell carcinoma^54^ HRG mRNA and ErbB3 protein levels were found to independently correlate with poor overall survival (OS), with the stronger effect coming from HRG. This combination of findings from us and others on HRG-ErbB3 activation in a variety of cancers is in many ways analogous to the pre-trastuzumab days with HER2 being a predictor of poor clinical prognosis. Thus, we suggest that the presence of HRG in tumors has broader implications when we consider the current clinical challenges in the precision medicine era. Namely, that HRG appears to be indicative of a novel cancer cell ‘phenotype’ characterized by increased proliferative and cell cycle rates and increased tumor survival under therapeutic pressures that ultimately leads to patients that are difficult to treat in comparison with those patients that do not exhibit persistence of HRG expressing cancer cells within their tumors. If one considers the heterogeneous nature of tumors, HRG-positive tumor cells exist side by side with their HRG-negative counterparts but once tumors are exposed to standard of care therapy it is the HRG-positive cells that persist, due their intrinsic drug-tolerant nature, and ultimately negatively impact clinical outcomes.

Collectively, these data all point to HRG mRNA being a clinically relevant biomarker indicative of poor clinical outcomes. Fortunately, HRG mRNA also appears to be an actionable biomarker. Blocking HRG with seribantumab appears to sensitize those cancer cells to concomitant therapy in essence converting them back to phenotypically HRG-negative cells. Interestingly, the PFS curves of HRG-positive patients receiving seribantumab plus standard-of-care closely resembles those of HRG-negative patients receiving standard-of-care alone (Figures 7c and d). This suggests that seribantumab acts primarily to block drug tolerance, rather than to inhibit a driver of tumor growth. A similar result was observed in the HERALD study, in which patritumab appeared to restore sensitivity to erlotinib in patients with high HRG levels.^52^ It remains to be seen if BTC emerges as an important biomarker for seribantumab in EGFR dependent cancers like colon or head and neck cancer.

The use of Systems Biology to highlight ErbB3 as a novel target, to specify the design criteria needed to inhibit this pathway effectively, and to uncover the biomarkers that define pathway activity in human tumors represents only the beginning of how the tools of computation and simulation can be used to focus and accelerate the process of drug discovery. The key to future success lies in learning how to translate insights obtained at the bench and *in silico* to the clinical setting. For example, Yaffe and coworkers recently showed how time-staggered dosing can be used to rewire signaling networks and unlock previously unrecognized drug synergies.^55^ Through mathematical modeling they were able to explain and predict complex interactions between growth factor signaling and the DNA damage response network. These types of insights have the potential to dramatically improve patient outcomes, but it will take innovative clinical trial designs and diagnostic strategies to test these ideas and reduce them to practice. As Systems Biology advances from the realm of basic science to clinical development, we may soon see a day in which modeling and simulation have an integral role not just in understanding complex biological phenomena, but in diagnosing and treating patients.

## Materials and methods

### Cell lines and NCI-60 screen

Cell lines were obtained from the NCI’s Developmental Therapeutics Program (http://www.dtp.nci.nih.gov) or purchased from American Type Culture Collection. All cell lines were maintained in RPMI 1640 medium (Gibco) supplemented with 10% fetal bovine serum (Hyclone Labs, South Logan, UT, USA), 2 mmol/l l-glutamine (Gibco), penicillin (100 U/ml), and streptomycin (100 mg/ml; Gibco) and propagated in a humidified atmosphere of 5% CO_2_ and 95% air at 37 °C. For the NCI-60 cell line screen, cells were plated to achieve approximately 75% confluence following proliferation in complete media for 24 h in 96-well plates and then starved in serum-free media for 24 h. Cells were exposed to growth factors (diluted and stored according to the manufacturer’s recommendation; Supplementary Table S1) at a final concentration of 100 ng/ml for 30 min and then lysed. The screen consisted of three technical replicates within each plate for each ligand stimulation and one experimental replicate (Supplementary Table S2). The quantitative p-Akt (S473) measurements were performed by enzyme-linked immunosorbent assay (ELISA) using a standard curve of recombinant protein to regress against. Total protein was measured by BCA assay (Pierce Biotechnology, Rockford, IL, USA). For each cell line, the median human serum albumin (HSA) control-based p-Akt signal was subtracted from the ligand-induced p-Akt signal and normalized to the maximum signal observed within each cell line.

Cell lines (HCC1419, BT474-M3, NCI-N87, AGS, ZR75-1, NCI-H358, ADRr-HER2 over-expressing, and OVCAR8) were seeded onto 96-well plates (Corning, CLS3550) in RPMI supplemented with 4% fetal calf serum, penicillin, and streptomycin. Twenty four hours post-seeding, Cells were stimulated with heregulin for 5 h (5 nmol/l, R&D Systems, Minneapolis, MN, USA) and then treated with seribantumab or MM-111, a bispecific antibody targeting HER2 and ErbB3 (0 or 1 μmol/l) for 2 h. Cells were washed with cold PBS and resuspended in lysis buffer (Invitrogen) supplemented protease and phosphatase inhibitors (Roche). P-ErbB3 levels were measured using a Luminex assay (ErbB3 capture antibody from R&D Systems, 4G10 detection antibody from Roche). Two independent experiments, each with two experimental replicates, were performed. Analysis was performed with GraphPad Prism software (http://www.graphpad.com/scientific-software/prism/). HER2 levels were determined by qFACs.^1^

MCF-7Ca (human breast cancer cells, which have been stably transfected with human aromatase gene) were provided by Dr S Chen (City of Hope, Duarte, CA, USA) in 2012 and maintained as previously described.^33^ A2780 and A2780cis cell lines was obtained from Sigma-Aldrich and maintained as described above. All cell lines were received prior to 2010 and were authenticated before receipt. Cells were propagated for less than 6 months after resuscitation and cultures were regularly tested for mycoplasma. Erlotinib and paclitaxel were purchased from LC Laboratories (Woburn, MA, USA).

### Enzyme-linked immunosorbent assay

To study intracellular signaling, cell lysates were prepared and ELISAs were performed as previously described.^1^ To generate tumor lysates, flash frozen tumors were pulverized in a CryoPrep pulverizer (Covaris, Woburn, MA, USA) and resuspended in T-PER Tissue Protein Extraction Buffer (Thermo Scientific, Tewksbury, MA, USA), supplemented with protease and proteinase inhibitors (Roche, Indianapolis, IN). HRG was detected by indirect ELISA as previously described.^1^

### Immunoblot analysis

For *in vitro* signaling studies, 50,000 cells were propagated overnight in 6 cm dishes followed by serum-starvation for an additional 20–24 h in media with 0.5% fetal bovine serum at 37 °C. Serum-starved cells were pre-incubated with either seribantumab, erlotinib, or a combination of both agents, followed by stimulation with either HRG, EGF or both ligands (R&D Systems). To generate cell lysates, cells were washed with ice-cold PBS followed by lysis with M-PER Mammalian Protein Extraction Buffer (Thermo Scientific, Tewksbury, MA, USA) supplemented with protease and proteinase inhibitors (Roche, Indianapolis, IN, USA). Total protein was measured using a BCA assay (Thermo Scientific). For immunoblot analyses, 30 μg protein was resolved by SDS-PAGE and various proteins and phospho-proteins were detected using fluorescently-labeled secondary antibodies. Bands were visualized using the Odyssey detection system (LI-COR, Lincoln, NE, USA) and quantitative analyses were performed using ImageStudio (LI-COR). All measurements were corrected by calculating ratios relative to a β-actin loading control. All antibodies were purchased from Cell Signaling Technology (Danvers, MA, USA), unless otherwise indicated: p-ErbB3 (#4784/ #4791), t-ErbB3 (#12708), p-Akt (#9271), t-Akt (#9272), p-ERK (#4370), t-ERK (#4370), β-actin (#3700).

### Spheroid growth assay and *in vitro* screening conditions

To measure cell viability in a three-dimensional spheroid culture, cells were seeded into 96-well low-binding multi-spheroid culture plates (Scivax USA) in culture medium supplemented with 4% fetal bovine serum, 1% penicillin and 1% streptomycin at a density of 5000 cells/well. To allow for spheroid formation, plates were incubated for 48 h, after which cells were treated with ligand (5 nmol/l HRG) and varying concentrations of inhibitors (seribantumab and/or chemotherapies) in medium containing 2% fetal bovine serum. Following 48 h of incubation, media, ligands and drugs were replenished, and the plates were incubated for an additional 48 h. Cell viability was determined by incubation with CellTiter-Glo (Promega Corporation, Madison, WI, USA) reagent for 10 min, with well luminescence measured using an Envision plate reader (Perkin Elmer, Shelton, CT, USA). Each cell line was tested separately for its ability to form spheroids in the nanoculture plates (with or without Matrigel (BD Biosciences, Bedford, MA, USA)) and its response to each chemotherapeutic drug at varying doses, in the absence or presence of 5 nmol/l HRG. This information was used to define appropriate dose ranges for each drug and each cell line.

### Seribantumab pharmacokinetic and pharmcodynamic study

Three to four week old female *nu/nu* mice were purchased from Charles River Laboratories (Wilmington, MA, USA). The care and treatment of experimental animals were in accordance with the Institutional Animal Care and Use Committee guidelines. Subcutaneous tumors were established by injecting five million A549 cells suspended in 1:1 RPMI 1640 media and growth factor reduced Matrigel into the right flank of recipient mice. When the average tumor volume reached approximately 200 mm^3^, mice were randomized into different treatment groups. For pharmacokinetic studies, mice received a single intraperitoneal (i.p.) dose of seribantumab at 150, 300, or 600 μg/mouse. Following 1, 4, 8, 24, 48, 72, 96, and 168 h after a single dose, mice were euthanized and serum was collected to determine seribantumab levels by ELISA. Samples were incubated for 2 h at room temperature in 96-well Maxisorb high-binding plates (Nunc,4737111) coated with recombinant His_6_-tagged ErbB3. Seribantumab was detected with HRP-mouse anti-human IgG (Invitrogen, cat#05–4220, Camarillo, CA, USA) for 2 h at room temperature. Luminescent signal was measured with SuperSignal ELISA Pico Chemiluminescent Substrate (Pierce, 37069). A one-compartment pharmacokinetic model with first order absorption and non-linear clearance was used to fit the observed serum concentration data in mice. For pharmacodynamics studies, mice received either one or two doses (every 3 days, i.p.) of seribantumab at 600 μg/mouse. Mice were euthanized 24, 48, and 72 h after dosing with seribantumab, and tumors collected to determine p-ErbB3 and t-ErbB3 levels by ELISA, as described above.

### Anti-tumor activity studies

Subcutaneous tumors were established in 3- to 4-week-old *nu/nu* mice by injecting either five million cells for A549 and H332M or two million cells for A2780 and A2780cis into the right flank of recipient mice. Changes in tumor volume (calculated using the following formula: volume=width^2^×length×0.52) were determined twice weekly by caliper measurement. When the average tumor volume reached approximately 200 mm^3^, mice were randomized into treatment groups. Seribantumab was administered via i.p. injections every three days at the indicated doses. For combination studies, erlotinib was administered orally every three days and paclitaxel was administered i.p. weekly at the indicated doses. The combination study with seribantumab and letrozole in MCF-7Ca xenografts has been described previously.^33^ The exponential tumor growth rate was quantified by log-transforming the tumor volume measurements and using a generalized linear mixed effect model.^56^ Fixed effect (time) and random effect (mouse ID) were used in order to get more robust estimates of tumor growth rate in each treatment group. Bliss additivity response^37^ was calculated based on percentage tumor growth rate inhibition, which can be defined as the changes in the exponential growth rate relative to the PBS control group:

$$\%\;\mathrm{Tumor}\;\mathrm{growth}\;\mathrm{rate}\;\mathrm{inhibition}\;\mathrm{of}\;\mathrm{treatment},\;i=\frac{\left(k_{PBS}-k_{i}\right)}{k_{PBS}}\times 100$$

where *k*_PBS_ and *k*_*i*_ are the exponential growth rate coefficients of the PBS control group and the treatment group, *i,* estimated from linear mixed effect models.

Efficacy studies using patient-derived xenograft models (MAX449, MAX1162, and MAX574) were conducted at Oncotest GmbH in Germany. The patient-derived xenograft tumor samples were obtained from Oncotest and HRG protein levels were measured by ELISA at Merrimack.

### Tissue sample analysis

Archived tissue and/or pre-treatment biopsies were acquired from each patient and five pre-specified biomarkers were measured in each sample: heregulin (HRG), betacellulin (BTC), EGFR, HER2, and ErbB3. All assays were performed using formalin-fixed, paraffin-embedded (FFPE) tissue sections. The protein levels of the receptors were measured either semi-quantitatively by chromogenic IHC assays or by quantitative IHC using fluorescence.^57^ For the quantitative IHC assay, fluorescence measurements in patient tumors were referenced to a standard curve, constructed using a tissue microarray of cell pellets comprising cell lines with known receptor levels (previously determined by quantitative FACS). Experimental details for these assays have previously been described (Liu J *et al.*, JCO in press).

### Computational model development and analysis

The computational model was constructed with the use of mass action kinetics describing ligand-induced ErbB receptor homo- and heterodimerization, receptor internalization and degradation, constitutive dimerization, binding of the downstream kinase PI3K, and subsequent activation of Akt and has been described in detail^1^ and can be downloaded from the BioModels database. This model was deposited in BioModels^58^ and assigned the identifier MODEL1609190001. The model was originally encoded for the MATLAB SimBiology Toolbox and was later exported to SBML. For comparison, the model also contains reactions describing inhibition of heregulin− and betacellulin-driven AKT signaling by MM-111 (a bispecific anti-ErbB2+anti-ErbB3 antibody) and erlotinib (an EGFR receptor tyrosine kinase inhibitor).Simulation conditions as described in the paper include two phases: an equilibrium phase followed by a treatment phase: In the equilibrium phase, levels of receptors (EGFR, ErbB2, ErbB3) are set to cell line-specific values and the model simulated for 30 min and in the treatment phase, inhibitor(s) and/or ligand(s) are added either sequentially or simultaneously (co-treatment).
